# Microtubule-Associated Protein SBgLR Facilitates Storage Protein Deposition and Its Expression Leads to Lysine Content Increase in Transgenic Maize Endosperm

**DOI:** 10.3390/ijms161226199

**Published:** 2015-12-12

**Authors:** Chen Liu, Shixue Li, Jing Yue, Wenhan Xiao, Qian Zhao, Dengyun Zhu, Jingjuan Yu

**Affiliations:** 1 State Key Laboratory for Agro-Biotechnology, College of Biological Sciences, China Agricultural University, No. 2 Yuanmingyuan West Road, Beijing 100193, China; liuchen@iae.ac.cn (C.L.); lishixue19890105@126.com (S.L.); yuejinglove@126.com (J.Y.); zb1106052@cau.edu.cn (W.X.); zhaoqian@cau.edu.cn (Q.Z.); zhudy@cau.edu.cn (D.Z.); 2 Key Laboratory of Pollution Ecology and Environmental Engineering, Institute of Applied Ecology, Chinese Academy of Sciences, No. 72 Wenhua Road, Shenyang 110016, China

**Keywords:** SBgLR, microtubule-associated protein, protein body formation, maize nutrient improvement

## Abstract

Maize (*Zea mays*) seed is deficient in protein and lysine content. Many studies have been made to improve the nutritional quality of maize seeds. Previously, we reported the role of a natural lysine-rich protein gene *SBgLR* in increasing protein and lysine content. However, how the SBgLR improves lysine and protein content remains unclear. Here, the reasons and possible mechanism for SBgLR in protein and lysine improvement have been analyzed and discussed. Through seed-specific expression of *SBgLR*, we obtained transgenic maize with the simultaneously increased lysine and protein contents. High-protein and high-lysine characters were stably inherited across generations. The expression of *SBgLR* in maize kernels increased the accumulation of both zeins and non-zein proteins. Transmission electron microscopy showed that the number of protein bodies (PBs) was increased obviously in SBgLR transgenic immature endosperms with the morphology and structure of PBs unchanged. The proteinaceous matrix was more abundant in transgenic mature endosperms under scanning electron microscopy. The stabilities of zein and lysine-rich non-zein genes were also increased in transgenic endosperms. Finally, the potential application of SBgLR in maize nutrient improvement was evaluated. This study shows that a cytoskeleton-associated protein has potential applicable value in crop nutrient improving, and provided a feasible strategy for improvement of maize grain quality.

## 1. Introduction

Maize is one of the most important cereals for the food and feed industry. It is an important source of proteins for humans and livestock, especially in developing countries [1,2]. Zein, the most abundant storage protein in maize kernels, is deficient in lysine and tryptophan, leading to a poor quality of protein for monogastric animals. Many approaches have been used to improve the nutritional quality of maize. *Opaque 2* (*o2*) is a naturally-occurring high-lysine mutant, which has twice the normal level of lysine and tryptophan [3]. However, this mutant has a soft endosperm [2]. The kernel softness is further overcome by several *o2* modifiers, and quality protein maize (QPM) has been developed by this approach [4]. In addition, a bacterial gene encoding a lysine feedback-insensitive dihydrodipicolinate synthase (DHDPS) has been introduced into maize to increase free lysine level [5]. Unfortunately, no lysine increase was observed when the gene was specifically expressed in starchy endosperm, even though more than a two-fold level of lysine accumulation was detected when expressed in the embryo and aleurone [5]. More recently, high-lysine maize has been obtained by decreasing lysine catabolism through RNA interference technique [6], by reducing α-zein mRNA [7,8], by expressing a milk protein α-lactalbumin [9], and by expressing a lysine-rich protein gene *GhLRP* in maize endosperm [10]. Except for some *GhLRP* transgenic lines, these high lysine corn plants contained normal or less level protein content in the seeds. However, seed-specific expression of lysine-rich protein gene *SB401* or its homolog *SBgLR* significantly increased both lysine and total protein contents in maize seeds [11,12,13]. Functional analysis showed that SB401 and SBgLR can bind and interact with the cytoskeleton and function in microtubule (MT) reorganizations [14,15]. But how these two genes improve protein and lysine content of transgenic maize seeds remains unclear.

It is known that zein accumulates in protein bodies (PBs) in the endosperm cells. However, the mechanism of PB formation, especially the role of the cytoskeleton system, is incompletely understood. Previous studies demonstrated that the cytoskeleton plays an important role in the spatial and temporal distribution of materials and information by affecting targeting, tethering, transporting and translation of mRNA [16,17,18]. In maize endosperm cells, extensive evidence showed that zeins are synthesized on cytoskeleton-bound polysomes [19,20] and their mRNAs were localized through binding to a specific site on the tubulin and actin cytoskeleton [21]. These data indicated that the cytoskeleton serves as an attachment scaffold for mRNA translation and protein synthesis [22,23]. eEF1α, a lysine-rich cytoskeleton-associated protein, plays an important role in protein synthesis and actin-bundling in maize endosperms [24,25,26]. PBs in endosperm cells are distributed between starch granules, where eEF-1α and cytoskeletal elements, such as actin filaments and microtubules (MTs), are abundant [27]. The eEF1α concentration was found to be a good predictor of the endosperm lysine content [28,29]. However, the roles of microtubule-associated proteins, and the relationship between the cytoskeleton system and storage protein accumulation are not fully understood.

The *SBgLR*, we cloned from *Solanum berthaultii*in previously [30], encodes a pollen-specific protein with high lysine content (18.93%, *w*/*w*) [12]. SBgLR contains five imperfectly repeated motifs of V-V-E-K-K-N/E-E, which resemble the repetitive domain responsible for MT-binding activity in murine and plants cells [15,31]. Considering these characteristics, we further investigated the activities of SBgLR on MT regulation. We found that the recombinant SBgLR binds to both tubulin and MTs *in vitro*. SBgLR overexpressing tobacco showed curving and right-handed twisting root growth; and abnormal directional expansion of cotyledon pavement cells, suggesting that SBgLR interacts with MTs and regulates their organization [15]. Our previous work showed that overexpression of *SBgLR* can increase the lysine and protein content in maize seeds [12,13]. In this research, we obtained SBgLR transgenic maize with the simultaneously increased lysine and protein contents in seeds and analyzed heredity of high-lysine and high-protein characters. In addition, through analyzing the SBgLR transgenic maize seeds by transmission electron microscopy (TEM) and scanning electron microscopy (SEM), we found that the expression of *SBgLR* promotes PB formation and proteinaceous matrix deposition. And transcription analysis showed increasing stabilities of storage protein genes and non-zein genes. The potential application of SBgLR transgenic maize lines was evaluated and the probable mechanism of SBgLR in improving the protein and lysine contents of maize seeds was discussed.

## 2. Results and Discussion

### 2.1. High-Protein and High-Lysine Characters are Stably Inherited in SBgLR Transgenic Lines

**Figure 1:**
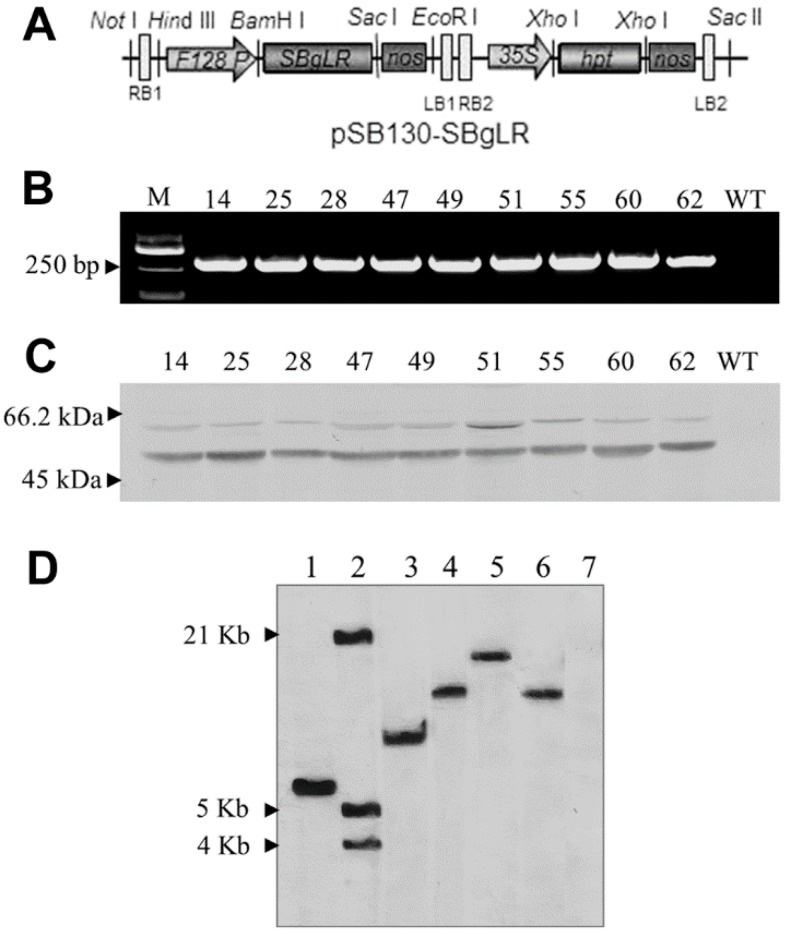
The diagram of binary vector and molecular analysis of transgenic plants. (**A**), Diagram of pSB130-SBgLR; (**B**), RT-PCR analysis of *SBgLR* transcript in transgenic line 14, 25, 28, 47, 49, 51, 55, 60, 62 and WT kernels. M, DNA marker; (**C**), Western blot analysis of SBgLR protein in transgenic line 14, 25, 28, 47, 49, 51, 55, 60, 62 and WT kernels. Two specific bands were detected from transgenic seeds protein, but not from WT protein; (**D**), Southern blot analysis of transgenic Q14 and Q51 plants. Lane 1, positive control. Lane 2, DNA marker, from bottom to top: 4 Kb, 5 Kb and 21 Kb respectively. Lane 3 and lane 4, Q14 genomic DNA digested with *Hind* III and *Bam*H I respectively. Lane 5 and 6, Q51 genomic DNA digested with *Hind* III and *Bam*H I respectively. Line 7, WT genomic DNA digested with *Hind* III as a negative control.

SBgLR was previously reported to increase lysine and protein content in T_1_ transgenic maize seeds [8,9]. In order to analyze the heredity of the high-protein and high-lysine characters and to obtain the SBgLR transgenic maize with the selection marker free, we introduced *SBgLR* gene into maize hybrid line Hi3027 (08 × 178) under the control of the seed-specific promoter *pF128* with the double “T-DNA” *Agrobacterium*-binary vector pSB130-SBgLR (Figure 1A) [32]. Finally, a total of 23 independent transgenic events were identified by polymerase chain reaction (PCR) amplification using *SBgLR*-specific primer pair S1/S2 (Table S1). Kernels of these plants were obtained by self-pollination. T_1_ plant genotyping showed that 14 transgenic lines exhibited segregation at a ratio of approximately 3:1, suggesting the presence of a single-site integration of the *SBgLR* gene. The expressions of the *SBgLR* gene in transgenic maize endosperm was detected by reverse transcription-polymerase chain reaction (RT-PCR). A 241-bp fragment was amplified from the coding region of the *SBgLR* gene in T_2_ immature kernels at 15 days after pollination (DAP) of each transgenic line, but not from wild type (WT) control (Figure 1B). This indicated that *SBgLR* was transcribed and spliced accurately in transgenic kernels as reported by Lang *et al.* [30]. Furthermore, Western blot was performed to investigate the accumulation of SBgLR protein in maize seeds. The result showed that two bands with different staining signals (weak and dark staining) were detected from transgenic seeds protein, but not from WT control (Figure 1C). Sequence analysis revealed that predicted glycosylation and phosphorylation sites existed in the SBgLR amino acid sequence (Figure S1). The weak staining band might be a post-translational modification form of SBgLR protein.

The protein and lysine content were analyzed in the seeds of T_1_ plants from 14 transgenic lines. Seeds of Hi3027 segregation progenies were used as a reference. The protein and lysine content of line 14, 25, 28, 47, 49, 51, 55, 60 and 62 were markedly increased (more than 20%) and were selected to sow in T_2_ generation (Table 1). We monitored the protein and lysine content of these transgenic lines in the continuous generations, the results showed that the high-lysine and high-protein characters could be stably inherited across generations (Table 1). Among all the transgenic lines, the protein content in T_2_ seeds of line 14 and 51 was 15.17 and 14.16 g/100 g dry weight, respectively, while it was 10.35 g/100 g dry weight in WT control (Table 1). Meanwhile, the lysine content in the seeds of these two lines was 0.49 and 0.44 g/100 g dry weight, respectively, and it was 0.28 g/100 g dry weight in WT control (Table 1). Southern blot further confirmed that a single copy of *SBgLR* expression cassette insertion in these two lines (Figure 1D) and the marker gene was not detected by PCR and Southern blot in these two lines (Data not shown). So these two lines named Q14 and Q51 were selected as materials for further study on how SBgLR increase protein and lysine content in transgenic maize seeds.

**Table 1:** Protein and lysine content in different transgenic lines.

Line	Protein Content (g/100 g) ^$^			Lysine Content (g/100 g) ^$^		
	T_1_	T_2_	T_3_	T_1_	T_2_	T_3_
14	14.21 ± 0.07 **	15.17 ± 0.84 **	15.20 ± 0.37 **	0.42 ± 0.03 **	0.49 ± 0.06 **	0.49 ± 0.03 **
25	12.78 ± 0.12 **	13.17 ± 0.96 **	13.05 ± 0.89 **	0.39 ± 0.02 **	0.38 ± 0.02 **	0.39 ± 0.02 **
28	14.26 ± 1.10 **	13.19 ± 1.48 **	13.67 ± 0.99 **	0.37 ± 0.02 **	0.34 ± 0.01 *	0.36 ± 0.01 **
47	12.72 ± 0.14 **	13.19 ± 1.04 **	13.34 ± 1.17 **	0.39 ± 0.01 **	0.39 ± 0.02 **	0.38 ± 0.03 **
49	12.61 ± 0.09 **	12.22 ± 0.10 **	12.99 ± 0.76 **	0.39 ± 0.01 **	0.41 ± 0.03 **	0.40 ± 0.01 **
51	14.29 ± 0.11 **	14.16 ± 1.07 **	14.37 ± 0.92 **	0.41 ± 0.03 **	0.44 ± 0.02 **	0.43 ± 0.01 **
55	12.60 ± 0.19 **	12.40 ± 0.28 **	13.94 ± 0.39 **	0.36 ± 0.03 **	0.35 ± 0.02 **	0.37 ± 0.01 **
60	12.59 ± 0.17 **	13.77 ± 0.46 **	13.17 ± 0.56 **	0.40 ± 0.02 **	0.42 ± 0.01 **	0.41 ± 0.02 **
62	12.46 ± 0.56 **	12.74 ± 0.79 **	13.41 ± 1.07 **	0.38 ± 0.01 **	0.39 ± 0.02 **	0.39 ± 0.01 **
CT ^$$^	10.54 ± 0.40	10.30 ± 0.88	10.78 ± 0.53	0.26 ± 0.01	0.28 ± 0.01	0.25 ± 0.01

Values shown are grams of protein or lysine per 100 g of kernel dry weight. Data were mean ± SD. ^$^, T_1_, T_2_ and T_3_ represents the plant generation. Data were collected from the seeds of T_1_, T_2_ and T_3_ plants. ^$$^, Hi3027 segregation progenies with the same generation as transgenic lines were used as control (CT). The data of protein and lysine content of control (CT) were the average value of twenty Hi3027 segregation progenies. Data were analyzed by One-way ANOVA (*p* < 0.05) and followed by Least Significant Difference (*LSD*) post test (* *p* < 0.05; ** *p* < 0.01).

### 2.2. Zeins And Non-Zein Proteins Accumulation in SBgLR Transgenic Maize Seeds

**Figure 2:**
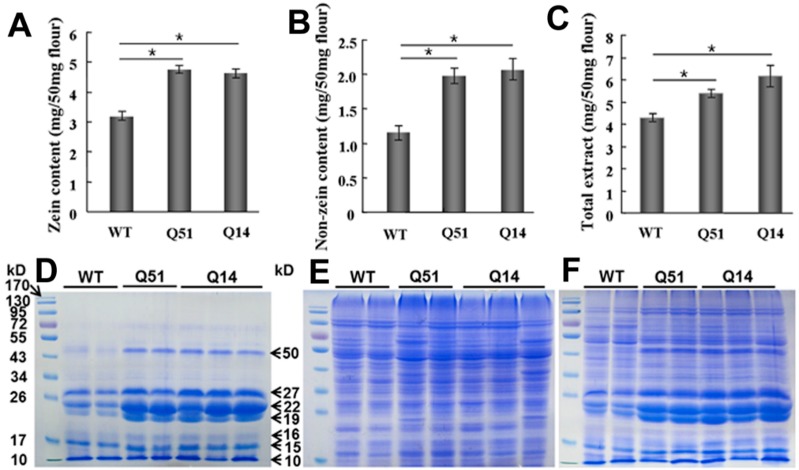
Analysis of protein accumulation in transgenic maize seeds. (**A**–**C**), Quantification assay of zein (**A**), non-zein (**B**) and total extract (**C**); (**D**–**F**), SDS-PAGE analysis of zein (**D**), non-zein (**E**) and total extract (**F**). The significance of difference was evaluated by Student’s *t*-test (* *p* < 0.05).

Maize storage proteins are mainly divided into two groups: zein and non-zein according to the differences of solubility. To explore which fractions of storage proteins were changed in transgenic kernels, we extracted and analyzed zein, non-zein and total protein extracts of the seeds of transgenic T_2_ and Hi3027 F_2_ plants by sodium dodecyl sulfate-polyacrylamide gel electrophoresis (SDS-PAGE) and protein content quantification assays. The results are shown in Figure 2. Total zein content of Q14 and Q51 mature seeds was 4.62 mg/50 mg and 4.75 mg/50 mg flour respectively, whereas it was 3.18 mg/50 mg flour of WT (Figure 2A). Except 10-kDa δ-zein, the contents of 15-kDa β-zein, 16-kDa γ-zein, 19-kDa α-zein, 22-kDa α-zein, 27-kDa γ-zein and 50-kDa γ-zein were all increased in transgenic mature seeds (Figure 2D). The non-zein proteins that contribute the most to maize lysine content were also increased in Q14 and Q51 mature seeds on SDS-PAGE staining gel (Figure 2E). They were increased to 2.06 mg/50 mg flour and 1.98 mg/50 mg flour in protein content quantification assay (Figure 2B). Total protein contents of WT, Q14 and Q51 were 4.27 mg/50 mg, 6.16 mg/50 mg and 5.38 mg/50 mg flour, respectively (Figure 2C,F). These results suggested that SBgLR is involved in both zeins and non-zein proteins enhancement in transgenic seeds.

### 2.3. Transmission Electron Microscopy of SBgLR-Overexpression Endosperm

**Figure 3:**
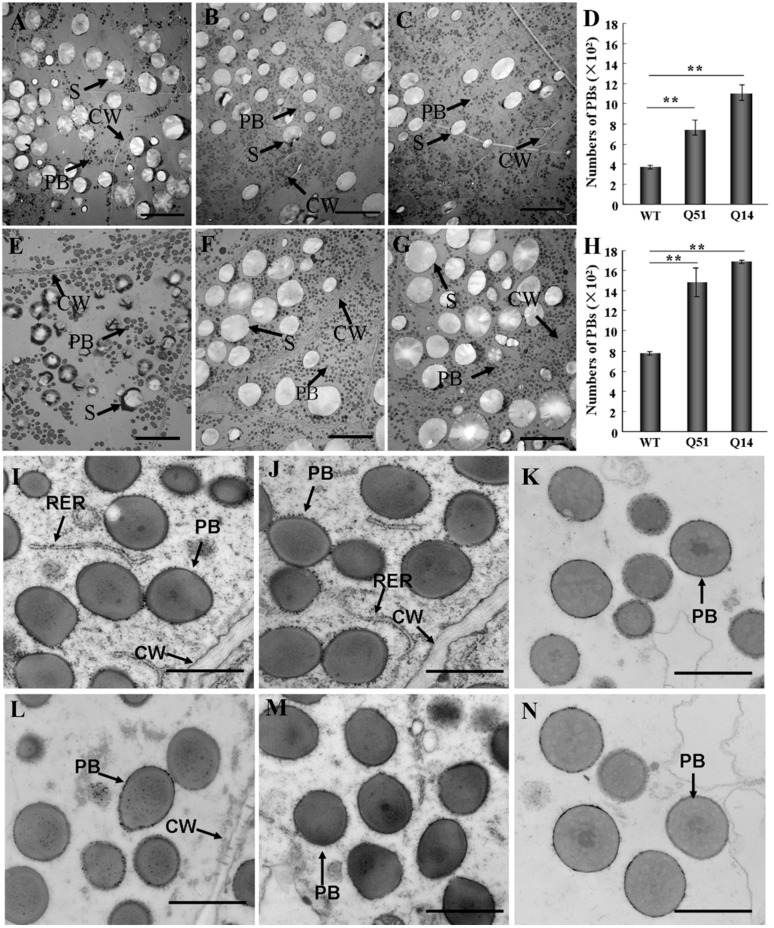
Transmission electron microscopy of 15 and 20 DAP endosperm cells. (**A**–**C**), Transmission electron microscopy of WT (**A**), Q14 (**B**) and Q51 (**C**) 15 DAP endosperm. Bar = 20 μm; (**E**–**G**), Transmission electron microscopy of WT (**E**), Q14 (**F**) and Q51 (**G**) 20 DAP endosperm. Bar = 20 μm; (**D**,**H**), Statistic of PB numbers per 60 μm^2^ micrograph in WT, Q14 and Q51 at 15 and 20 DAP endosperms. Data were mean ± SD, (** *p* < 0.01, Student’s *t*-test); (**I**–**K**), PB morphology observation of Q14 (**I**), Q51 (**J**) and WT (**K**) at 15 DAP endosperm; (**L**–**N**), PB morphology observation of Q14 (**L**), Q51 (**M**) and WT (**N**) at 20 DAP endosperm. Bar = 200 nm in (**I**–**N**). RER, rough endoplasmic reticulum; CW, cell wall; S, starch granule.

PBs are the major storage form of different types of zeins in maize endosperm. The composition of zeins in PBs has a decisive effect on PB morphology [33]. We have shown that zeins were markedly increased in transgenic endosperm cells. But how are these proteins stored in the endosperm cells? Two possibilities exist. One is that the increased zeins deposited into PBs form larger ones; and the other is that these zeins form more PBs in endosperm cells. To investigate the storage form of the increased zeins in SBgLR transgenic endosperm, several cell layers of Q14 and Q51 endosperm from T_2_ plants at 15 DAP and 20 DAP were analyzed with TEM. Endosperm samples from WT kernels (Hi3027 F_2_ segregation progeny) were used as references. Compared with WT (Figure 3A), the PBs were more abundant in Q14 and Q51 endosperms at 15 DAP and 20 DAP (Figure 3B,C). Quantitative analysis showed that the number of PBs in Q14 and Q15 endosperms was significantly greater than that in WT control (Student’s *t*-test, *p* < 0.01). In WT endosperm cells at 15 DAP, the average number of PBs per 60 μm^2^ micrograph was 379.54 ± 18.41, while it was 1115.20 ± 39.18 and 952.37 ± 32.67, respectively, in Q14 and Q51 endosperm cells (Figure 3D). Similarly, the number of PBs was increased significantly in Q14 (Figure 3F) and Q51 (Figure 3G) endosperm cells at 20 DAP. They were 2.16 and 1.91 times of that in WT control, respectively (Figure 3H). Morphology observation showed that the size and shape of PBs were the same in Q14 (Figure 3I) and Q51 (Figure 3J) endosperm cells as in WT references (Figure 3K) at 15 DAP. As the seed develops, PBs increase in size in WT endosperm cells (Figure 3N) whereas almost not changed in Q14 (Figure 3L) and Q51 (Figure 3M) endosperm cells. These results indicated that the expression of SBgLR increases the number of PBs in transgenic endosperm cells.

**Figure 4:**
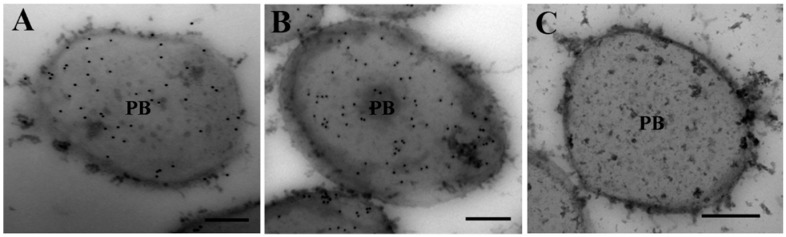
Immunogold labeling of 19-kDa α-zein. (**A**), WT; (**B**), Q14 20 DAP endosperm; (**C**), Primary antibody was omitted as negative control. Bar = 200 nm in (**A**–**C**). PB, protein body.

As the localization of zeins within PBs is important in the morphology of PBs [34], we conducted immunogold staining assays using antibody against 19-kDa α-zein; it was mainly, but not entirely, distributed over the light-staining region of PBs in WT (Figure 4A) cells. A similar distribution of 19-kDa α-zein were observed in Q14 endosperm cells (Figure 4B), suggesting a normal localization of zeins within PBs. No particle was detected when primary antibody was omitted in WT control (Figure 4C). These results indicate that SBgLR does not affect the localization of zeins in PBs.

### 2.4. Scanning Electron Microscopy of Transgenic Mature Endosperm

**Figure 5:**
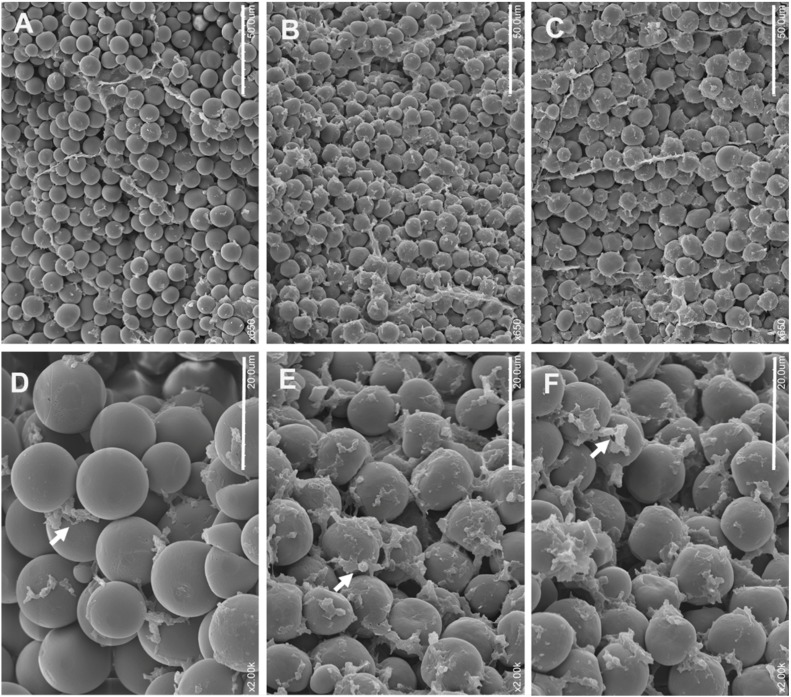
Scanning electron microscopy of mature seeds starchy endosperm (central region). (**A**,**D**), WT mature endosperm; (**B**,**E**), Transgenic Q14 mature endosperm; (**C**,**F**), Transgenic Q51 mature endosperm. Bar = 50 μm in (**A**–**C**); Bar = 20 μm in (**D**–**F**). White arrows indicate proteinous matrix.

TEM results showed that the number of PBs was increased in SBgLR transgenic immature endosperm cells. We further observed the proteinaceous matrix deposition of SBgLR transgenic mature seeds under SEM. The proteinaceous matrix mainly consisted of zein and non-zein proteins, and was located between starch granules. We examined the central region of the mature starchy endosperm where the deposition of proteinaceous matrix between starchy grains was easily observed under SEM. In WT mature kernels, endosperm cells in the central region of the starchy endosperm contained smooth starch grains with little proteinaceous matrix (Figure 5A,D). However, greater amounts of proteinaceous matrix were accumulated surrounding the spheroidal starch granules in Q14 (Figure 5B,E) and Q51 (Figure 5C,F) mature starchy endosperms. Observation at higher magnification revealed that proteinaceous matrix surrounding starch grains was more dense compared to that in WT endosperm (white arrow in Figure 5D), and distributed consecutively in Q14 (white arrow in Figure 5E) and Q51 endosperms (white arrow in Figure 5F) .

### 2.5. mRNA Stability Analysis of Zein and Lysine-Rich Non-Zein Genes in Transgenic Endosperm Cells

**Figure 6:**
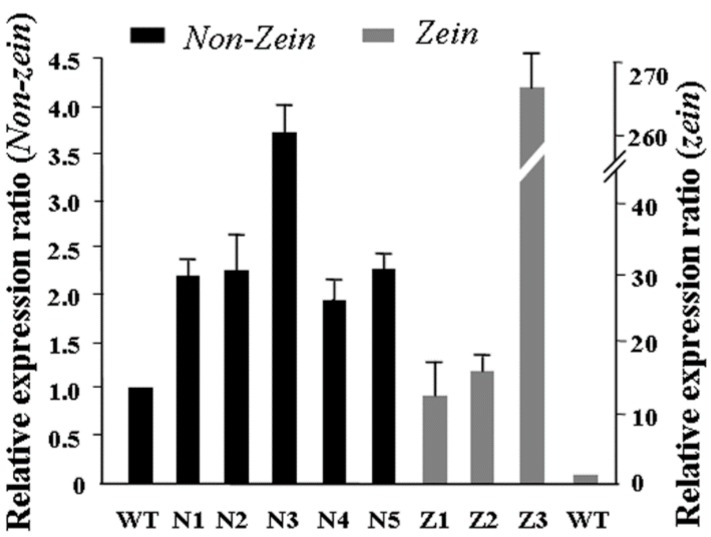
Quantitative analysis of some *zein* and lysine-rich *non-zein* transcripts in 20 DAP endosperm. N1, *histone H2B.3* (BM080549); N2, *cylicin-1* (CF627896); N3, *ER lumen protein retaining receptor C28H8.4* (CF602623); N4, *fructose-bisphosphatealdolase* (CF624216); N5, *60S ribosomal protein L22-2* (BI543125); Z1, 19 kDa *α zein* (AF371268); Z2, 22 kDa *α zein* (AF371277.1); Z3, *δ zein storage protein gene* (AF371265.1). Maize *actin* (LOC100280540) was used as reference gene.

We have shown that zein and non-zein protein accumulation were enhanced in transgenic endosperm cells. In order to investigate the differences of mRNA levels between transgenic and WT endosperm cells, a gene chip assay was performed using WT and Q51 20 DAP endosperm. The result showed that, compared to WT reference, the mRNA levels of some zein and lysine-rich non-zein protein genes were higher in Q51 immature endosperm cells. This primary result was further confirmed by quantitative RT-PCR. The expression level of maize 19-kDa α *zein* (AF371268), 22-kDa α *zein* (AF371277.1) and δ *zein storage protein gene* (AF371265.1) in transgenic endosperms were 12.01, 16.08, and 269.86 times higher, respectively, than in WT endosperms (Figure 6). Meanwhile, the expression level of five non-zein genes, *histone H2B.3* (BM080549), *cylicin-1* (CF627896), *ER lumen protein retaining receptor* C28H8.4 (CF602623), *fructose-bisphosphatealdolase* (CF624216) and *60S ribosomal protein* L22-2 (BI543125), which encode lysine-rich proteins, were 2.68, 2.21, 3.71, 1.95 and 2.28 times higher, respectively, than in WT endosperms (Figure 6). This result indicated that the mRNA stabilities of storage protein genes were also increased in SBgLR transgenic endosperm cells.

### 2.6. Potential Application of Transgenic Maize Lines in Nutrient Maize Breeding

**Figure 7:**
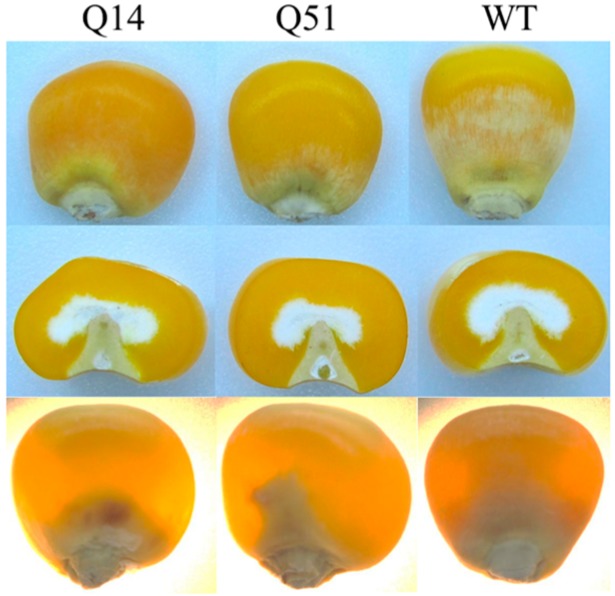
Kernel phenotype for Q14, Q51 and WT. Photographs of kernels were taken under incandescent light (**Top** and **Middle**) or with transmitted light (**Bottom**).

High protein and high lysine maize inbred lines are valuable in maize nutrient improvement. To evaluate the potential application of SBgLR maize, kernel quality and agronomic traits were analyzed. Kernel hardness, a critical agronomic trait, is very important in maize breeding. Under incandescent and transmitted light, the kernels of T_5_ generations of Q14 and Q51 showed the vitreous phenotypes as well as WT (Figure 7). No obvious differences of seed weight and kernel density were found (Figure S2A,B). Agronomic characters such as plant height, ear height, ear length, kernel weight per ear and ear rows were not affected in different generations of these transgenic lines (Tables S2–S4). Other nutrients such as starch and oil are also important. We analyzed the starch and oil contents of the seeds of T_1_, T_4_ and T_5_ transgenic plants. The data shown in Table 2 indicate that starch and oil content were not changed significantly in these transgenic lines (Table 2). Furthermore, data from our field test showed that the germination rates of T_2_ seeds of these two lines was also similar to that of the WT (Figure S2C). These results indicate that the increase of protein and lysine contents in transgenic lines did not affect the agronomic characters and kernel qualities and these transgenic maize lines are potentially promising for corn breeding.

**Table 2:** Starch and oil content of the seeds of T_1_, T_4_ and T_5_ plants.

Line	Oil Content (g/100 g)			Starch Content (g/100 g)		
	T_1_	T_4_	T_5_	T_1_	T_4_	T_5_
14	4.66 ± 0.04	4.89 ± 0.59	4.49 ± 0.69	65.76 ± 0.40	64.34 ± 1.01	64.02 ± 1.60
25	3.94 ± 0.32	4.35 ± 0.20	4.13 ± 0.47	66.55 ± 0.22	65.72 ± 0.40	65.43 ± 1.09
28	4.02 ± 0.37	4.75 ± 0.86	4.66 ± 0.83	66.25 ± 0.61	68.10 ± 2.25	69.61 ± 0.72
47	5.45 ± 0.26	4.95 ± 0.38	4.60 ± 0.15	67.72 ± 0.92	67.85 ± 0.76	66.18 ± 0.67
49	4.65 ± 0.30	4.20 ± 0.32	4.42 ± 0.82	67.27 ± 0.70	68.51 ± 0.97	68.81 ± 1.01
51	5.87 ± 0.31	5.26 ± 0.26	5.13 ± 0.64	65.50 ± 0.27	67.41 ± 0.65	68.41 ± 2.65
55	5.22 ± 0.78	5.12 ± 0.28	5.42 ± 0.41	65.45 ± 0.84	66.45 ± 0.94	66.95 ± 1.17
60	5.08 ± 0.34	5.03 ± 0.14	5.13 ± 0.33	65.26 ± 0.68	66.39 ± 1.17	67.57 ± 0.54
62	5.27 ± 0.50	5.06 ± 0.30	4.92 ± 0.34	63.09 ± 0.76	67.28 ± 1.16	67.35 ± 0.64
CT	4.15 ± 0.58	4.34 ± 0.31	4.41 ± 0.60	65.60 ± 0.94	67.12 ± 0.54	67.32 ± 0.91

CT, Hi3027 segregation progenies. Data are mean ± SD.

### 2.7. Discussion

Protein and lysine content are the most important nutritional characters in maize. Overexpression of *SBgLR* can increase the lysine and protein content in maize seeds [12,13]. Here, we analyzed the protein and lysine content of different generations of *SBgLR* transgenic maize seeds and showed that the high-lysine and high-protein content characters could be stably inherited across generations (Table 1). Additionally, kernel quality and agronomic traits of transgenic lines were as normal as WT control (Figure 7, Table 2 and Tables S2–S4). This indicated that SBgLR transgenic maize had potential use in maize breeding for nutritional improvement.

Further, we analyzed the reasons why SBgLR increased protein and lysine content. The result showed that the expression of SBgLR increased both zein and non-zein proteins in transgenic seeds (Figure 2). Further observations through TEM and SEM indicated that the number of PBs and proteinous matrix was significantly increased in SBgLR transgenic endosperm (Figure 3 and Figure 5), while, the morphologies of PBs and zein localizations in PBs were not affected (Figure 3I–N and Figure 4).

The cytoskeleton plays an important role in storage protein accumulation and PB formation. Zeins are synthesized on cytoskeleton-bound polysomes and their mRNA localize by binding to a specific site on the tubulin and actin [19,20]. PBs in developing maize endosperms are surrounded by an extensive cytoskeletal network through which zeins are deposited into them [35]. In the *o2* mutant, the morphology of PBs is abnormal, and the cytoskeleton matrix is loose and unstable simultaneously [33]. Therefore, storage protein synthesis and PB formation are highly correlated and dependent on cytoskeleton activity. Many studies have demonstrated that the activity and organization of the cytoskeleton is regulated by cytoskeleton-associated protein [36]. For example, elongation factor 1α (EF-1α), a lysine-rich protein, bundles microfilaments and co-localizes with actin surrounding PBs to facilitate zein peptide elongation [27,37]. Our previous work showed that SBgLR binds and bundles MTs *in vitro*, and has an effect on MT organization, indicating SBgLR is a microtubule-associated protein [15]. The expression of SBgLR in tobacco root epidermal cells leads to curving and twisting root growth, abnormal cell expansion and cell layer arrangement [15]. In this study, a preliminary immunogold labeling assay showed that SBgLR was mainly detected at the periphery of PBs (white arrows in Figure S3A) and cytoskeleton-like structures surrounding PBs in transgenic maize endosperm (white arrows in Figure S3B). This result suggested the possible role of SBgLR in MT organization in endosperm cells, and that increased protein and lysine content is correlated with its cytoskeleton regulation activity. When storage proteins begin to accumulate, MTs and microfilaments organize into a meshwork and serve as attachment sites for protein synthesis and PB formation [20,21,22]. We hypothesize, in transgenic endosperm, SBgLR interacts with MTs and regulates their organization, and that a more stable and more compact cytoskeleton system compared with the WT is established, which promotes mRNA stability; this may explain why *zein* and *non-zein* transcripts were increased in transgenic endosperm (Figure 6). Consequently, the translation efficiency is increased, leading to more zein and non-zein protein accumulation in transgenic endosperm (Figure 2). The increased zeins were further deposited into PBs, increasing the number of PBs in transgenic endosperm (Figure 3A–H). Though the efficiency of storage protein synthesis increases in transgenic endosperm, the accumulation of zein and non-zein proteins cannot increase to unlimited levels, possibly because the soil nutrients, especially nitrogen supplementation, are relatively limited. This is perhaps the most reasonable explanation why the number of PBs was increased, but the size of PBs was almost unchanged in transgenic endosperm (Figure 3I,L,J,M). Some evidence indicates that cytoskeleton-associated non-zein proteins, such as carbohydrate-metabolizing enzymes (CHMEs) and EF-1α, make a significant contribution to the lysine content of corn [29,38]. Therefore, non-zein proteins together with the accumulation of SBgLR should contribute to the increased lysine content in transgenic maize. The discussion above provides a possible explanation for the role of SBgLR in increasing protein and lysine content in an indirect manner. However, further studies are needed to confirm the function and clarify the mechanism of SBgLR in transgenic maize seeds.

## 3. Experimental Section

### 3.1. Plasmid Construction and Maize Transformation

To construct the binary vector pSB130-SBgLR for maize transformation, the *SBgLR* (GenBank: AY377987.1) expressing cassette was sub-cloned into *Hind* III and *Eco*RI sites of pSB130, which was kindly provided by Siwen Xin (Chinese University of Hong Kong, Hong Kong, China) (Figure 1A).

To obtain transgenic maize lines, two elite maize inbred lines 08 and 178 were pollinated as female and male for the production of hybrid Hi3027. Immature embryos of the maize hybrid Hi3027 (08 × l78) were used as explants, and *Agrobacterium*-mediated transformation was performed as in a previously described method [39].

### 3.2. PCR Amplification and Southern Blot Hybridization

The presence of transgene in regenerated plants was confirmed by PCR amplification. The PCR was carried out using 50 ng DNA and the primer sequences (S1/S2) used in the reactions are listed in Table S1. The amplification parameters were set as follows: 95 °C for 5 min, 94 °C for 1 min, 56 °C for 1 min, 72 °C for 1 min, repeat for 30 cycles and a final extension for 10 min at 72 °C. Reactions were carried out with Taq DNA polymerase (Tiangen, Beijing, China) in a Biometra Thermo cycler (TG-96; Biometra, Horsham, PA, USA), and the PCR products were analyzed on 0.8% agarose gels.

For Southern blot hybridization, genomic DNA was isolated from fresh leaves using a cetyltrimethyl ammonium bromide (CTAB) method [40]. A total of 30 μg genomic DNA was digested with *Hind* III or *Bam*H I restriction enzyme (TaKaRa, Tokyo, Japan) and electrophoresed on 0.8% agarose gel and blotted onto Hybond TM-XL membrane (Amersham, Piscataway, NJ, USA). Hybridization was carried out according to the manufacturer’s protocol of Dig High Primer DNA Labeling and Detection Starter Kit I (Roche, Penzberg, Germany).

### 3.3. RT-PCR and Western Blot Detection

Immature kernels of T_2_ plants of each line at 20 DAP were randomly selected for genotyping. Embryos were separated for DNA extraction, and kernel genotype was determined by PCR using primer pair S1/S2 (Table S1). Each transgenic endosperm was cut into two halves, one for RNA extraction and the other for protein extraction, which were used for RT-PCR and Western blot analysis respectively.

Total RNA of immature endosperm at 20 DAP was extracted using TRIzol reagent (CWBIO, Beijing, China). The cDNA first strand synthesis was carried out according to the manufacturer’s protocol of Reverse Transcription System (Promega A3500, Fitchburg, WI, USA). RT-PCR amplification was performed using SBgLR (ORF) specific primers (SBRT) (Table S1) and carried out according to the procedure described above.

For Western bolt analysis, half of the 20 DAP endosperm was ground in 500 μL extraction buffer (20 mM Tris-HCl, pH 8.0, 5 mM EDTA, 0.05% SDS, 10 mM DTT, 1 mM PMSF) and vortexed at 4 °C for 1 h, then centrifuged at 4 °C for 20 min and the suspension was used for Western blot detection. In this study, 20 μL samples were separated on 12% SDS-PAGE and transferred to PVDF membranes (Millipore Corporation, Billerica, MA, USA). The membranes were blocked (3% BSA in TBST, 50 mM Tris-HCl, 150 mM NaCl, 0.05% Tween-20, pH 7.5) at 4 °C overnight and incubated with anti-SBgLR antibody (1:1000) for 1 h. Alkaline phosphatase-conjunct goat anti-rabbit IgG antibody was added (1:5000) after washing the membrane 15 min for three times. The hybridization signal was detected by using NBT/BCIP reaction kit (Promega, S380C, S381C, Fitchburg, WI, USA).

### 3.4. Agronomic Quality Measurement

To analyze the kernel density, 100-kernel-weight and volume was measured. Germination rate was calculated according to the statistical data obtained from our field test. Forty-five seeds were sowed in the field test and the germination rate was the ratio of germinated seeds to total seeds. The starch and oil content of mature seeds were analyzed by Fourier transform near-infrared (FT-NIR) spectrometer VECTOR22/N (BRUKER, Bremen, Germany).

### 3.5. Protein, Lysine, Zein and Non-Zein Contents Analysis

About 5 g of mature kernels were dehydrated in electric thermostatic drying oven at 80 °C for 48 h. The dehydrated kernels were ground into powder. Total nitrogen of kernels were determined by a nitrogen detection apparatus based on the principle of Kjeldahl determination using national standard GB2905-82 at Beijing Academy of Agriculture and Forestry Science (Beijing, China). The nitrogen content was converted to protein content by multiplying a conversion factor of 6.25.

Lysine content was analyzed based on the principle of ninhydrin reaction. Briefly, about 10 mg of defatted powder, 1 mL ddH_2_O and 2 mL ninhydrin reaction reagent were added into a 30-mL tube, and then the mixture was thoroughly vortexed. Subsequently, the tube was incubated in boiling water for 20 min, and 3 mL 50% ethanol was added to the cooled sample, followed by a centrifugation at 13,400× *g* for 10 min. Supernatant was collected, and the absorbance at 570 nm was recorded using a spectrophotometer (TECHCOMP UV2300, Beijing, China). Standard curve was conducted using a series concentration of leucine solution (from 0 to 50 μg/mL). Lysine content of each sample was calculated using the equation as follows: Lys content (g/100 g dry weight) = (measured lysine content × hydrolysis volume)/sample weight.

Zein and non-zein proteins were extracted from 50 mg endosperm flour according to the previously described method [41]. A total of 200 μL ddH_2_O was added to each fraction for SDS-PAGE and protein content analysis. Quantification of total extract, zein and non-zein fractions were performed using a BCA protein assay kit (Pierce, Appleton, WI, USA). For SDS-PAGE, 10 μL samples were loaded and electrophoresed in 12% polyacrylamide gel, and the gel was stained with Coomassie brilliant blue R250 (Amresco, Solon, OH, USA).

For statistical analyses, One-way ANOVA (*p* < 0.05) was performed to evaluate the difference between the measured traits. The significance of difference between WT and the transgenic lines was evaluated by *LSD* post-test (* *p* < 0.05; ** *p* < 0.01). All statistical analysis were made using the “Data Analysis” function in Microsoft Excel 2010 (Microsoft, Redmond, WA, USA).

### 3.6. Quantitative RT-PCR Analysis

For quantitative RT-PCR, embryos taken from single 20 DAP kernels were used for genotyping. Total RNA was extracted from the endosperm harboring the transgene using TRIzol reagent (CWBIO). The cDNA first strand synthesis was carried out according to the manufacturer’s protocol of Reverse Transcription System (Promega A3500, Fitchburg, WI, USA). Quantitative RT-PCR was performed in a 20-μL reaction system containing SYBGREEN reaction mixture (Takara, Japan), 10 μM of each primer and 50 ng cDNA. The sequences of the primers, δ-zein for *δ storage protein zein* *gene* (AF371265.1), 19-zein for 19 kDa *α zein* (AF371268), 22-zein for 22 kDa *α zein* (AF371277.1), histone H2B for *histone H2B.3* (BM080549), cylicin for *cylicin-1* (CF627896), ER protein for *ER lumen protein retaining receptor C28H8.4* (CF602623), FBA for *fructose-bisphosphatealdolase* (CF624216) and Ribosomal protein for *60S ribosomal protein L22-2* (BI543125), are listed in Table S1. Amplification was carried out on a Bio-Rad Real-Time System CFX96TM C1000 thermal cycler (Bio-Rad, Waltham, MA, USA) using the following conditions: 95 °C for 30 s, 35 cycles of 95 °C for 10 s, 60 °C for 10 s and 72 °C for 10 s. Maize *actin* (Table S1) was used as reference gene. Expression levels of these genes were determined as the *C*t values [42]. The *C*t values were then converted into relative quantities.

### 3.7. Transmission Electron Microscopy (TEM)

The presence of a transgene was detected by PCR using the primer pair S1/S2 as described previously. The transgenic endosperms with the thickness of 2-mm from T_2_ plants were perpendicularly sectioned to the pericarp, including the aleurone and 10 to 20 cell layers of endosperm. The slices were fixed in freshly prepared potassium phosphate buffer (50 mM, pH 6.8) containing 1% glutaraldehyde, 4% paraformaldehyde and 5 mM EGTA at 4 °C overnight. The fixed slices were rinsed at room temperature for 2 h, post-fixed with fixation buffer containing 1% osmium tetroxide at 4 °C overnight and dehydrated in gradient ethanol. The tissues were embedded in Spurr and LR White resin for transmission electron microscopy (TEM) or immunogold labeling assay, respectively. Plastic blocks were sectioned into 90 nm including six to eight cell layers, and collected on formvar-coated nickel grids. Non-transgenic endosperm segregated from transgenic line was used as negative control.

For TEM, grids were stained with 2% uranyl acetate and 2.66% lead citrate, followed by three rinses in ddH_2_O. The stained grids were air-dried and observed under a Hitachi 7500 electron microscope (Hitachi, Japan) operated at 80 kV. Pictures were taken using ITEM (Olympus, Tokyo, Japan). For PBs numbers quantification, data were collected from the measurement of at least 30 cells from two different kernels from two individual ears. Stutent’s *t*-test was performed between WT and two transgenic lines respectively to evaluate the significance of differences (* *p* < 0.05; ** *p* < 0.01).

For immunogold labeling assay, grids were blocked in blocking buffer (3% BSA, 0.05% Tween-20 in 50 mM potassium phosphate buffer, pH 6.9) and incubated with primary antibody (1:200) (the anti-19 kDa α-zein antibody was kindly provided by Brian A. Larkins) at 4 °C overnight. Subsequently, the grids were incubated with gold-conjugated secondary antibody at room temperature for 1 h and rinsed in ddH_2_O for three times. The grids were then stained as described above. The stained grids were examined with a Hitachi 7500 transmission electron microscope (Hitachi, Tokyo, Japan). Pictures were taken using ITEM (OSIS, Germany).

### 3.8. Scanning Electron Microscopy (SEM)

Mature seeds of T_2_ transgenic and Hi3027 F_2_ plants were dissected from the crown to the base and mounted on the surface of a brass disk using double-sided adhesive silver-tape. They were then coated with gold/palladium using an ion coater (EIKO IB.3, Tokyo, Japan), and the central region of starchy endosperm was examined using SEM (JEOL, Tokyo, Japan).

## 4. Conclusions

We analyzed SBgLR transgenic maize with high-lysine and high-protein content in seeds. Both PB formation and proteinaceous matrix deposition were promoted. The increased accumulation of zeins and lysine-rich non-zein proteins were the reason for protein content increment. Further, the increased deposition of non-zein protein, as well as the accumulation of SBgLR, contributes to the lysine increment in transgenic endosperm. The high-lysine and high-protein traits are stably inherited across generations. SBgLR can be considered for use in improving nutrient quality of maize seeds. This work indicates that a cytoskeleton-associated protein has potential applicable value in improving crop seed nutrient quality and provides a feasible strategy for maize grain quality improvement.

## Supplementary Materials

Supplementary materials can be found at http://www.mdpi.com/1422-0067/16/12/26199/s1.
