# Supplementary material for: Microtubule-Associated Protein SBgLR Facilitates Storage Protein Deposition and Its Expression Leads to Lysine Content Increase in Transgenic Maize Endosperm
Source: Int J Mol Sci. 2015 Dec 12;16(12):29772–86. doi: 10.3390/ijms161226199 (PMC4691142; doi:10.3390/ijms161226199)
Supplement: Supplementary file 1 [file ijms-16-26199-s001.docx]

Supplementary Materials: Microtubule-Associated Protein SBgLR Facilitates Storage Protein Deposition and Its Expression Leads to Lysine Content Increase in Transgenic Maize Endosperm

Chen Liu, Shixue Li, Jing Yue, Wenhan Xiao, Qian Zhao, Dengyun Zhu and Jingjuan Yu

**Table S1.** List of primer sequences.

| **Primer** | **Sequences** |
| --- | --- |
| S1/S2 | Forward: 5′-GAGAACGCTACCATTCCTAA-3′ |
|  | Reverse: 5′-GTGAGTCATTGACAGCCCTA-3′ |
| SBORF | Forward: 5′-CTGATATCATGGGTTGTGGGGA-3′ |
|  | Reverse: 5′-GGCTCGAGCTAATAAACACTCT-3′ |
| SBRT | Forward: 5′-GAAAGTGGTGGCTGTGGAAA-3′ |
|  | Reverse: 5′-AATTGGCTTGATGGTCTCCT-3′ |
| 18-zein | Forward: 5′-ATGGTGCCGACTATGATGTC-3′ |
|  | Reverse: 5′-ACAAAGGGCTGCTGTAAAAA-3′ |
| 19-zein | Forward: 5′-TTCACATACCGAAGACACCGC-3′ |
|  | Reverse: 5′-GGTCTTCAGACCATTAGCTTTATCT-3′ |
| 22-zein | Forward: 5′-TAGTTGTGTTGCGATGGTTTGT-3′ |
|  | Reverse: 5′-CGCTCCTTTCCCTTTCAGTGAG-3′ |
| Cylicin-1 | Forward: 5′-CCTTCCCTGAATCCTCACCACC-3′ |
|  | Reverse: 5′-AAGGTCAAGAGCAAGGACGAGC-3′ |
| Histone H2B | Forward: 5′-GAGGACGAGGCGCACCGAGGTC-3′ |
|  | Reverse: 5′-AGGGCAAGAAGGGGAAAAAGAA-3′ |
| ER protein | Forward: 5′-AACACCACATAATACAACGCAA-3′ |
|  | Reverse: 5′-TCCTCATCTACAAGCTAACCAA-3′ |
| Ribosomal protein | Forward: 5′-CGCCTATTCCTCCTCGTTCTCA-3′ |
|  | Reverse:5′-AGTCCAAGGTCACCGTCACCTC-3′ |
| FBA | Forward: 5′-GGAGATGAGCTCGTCCGAGTAGGAG-3′ |
|  | Reverse: 5′-ACCAACCCACCTCCTAGTTCCTGTT-3′ |
| Actin | Forward: 5′-CGTGTTGGATTCTGGTGATG-3′ |
|  | Reverse: 5′-AGCCACATATGCGAGCTTCT-3′ |


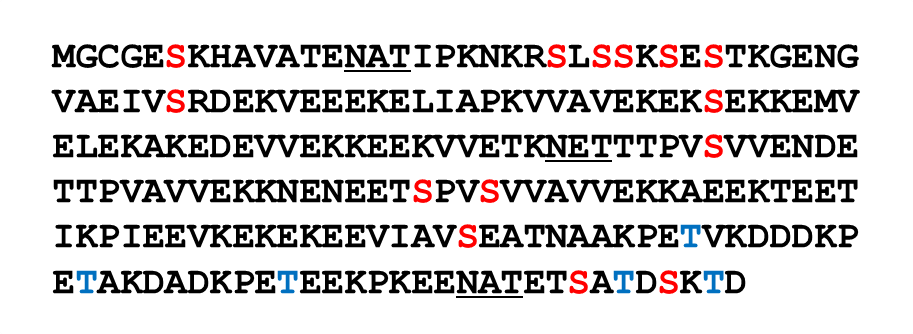


**Figure S1.** Amino acids sequence of SBgLR protein. Possible glycosylation sites were marked using underline tag; possible serine and threonine phosphorylation sites were marked in red and blue characters respectively.


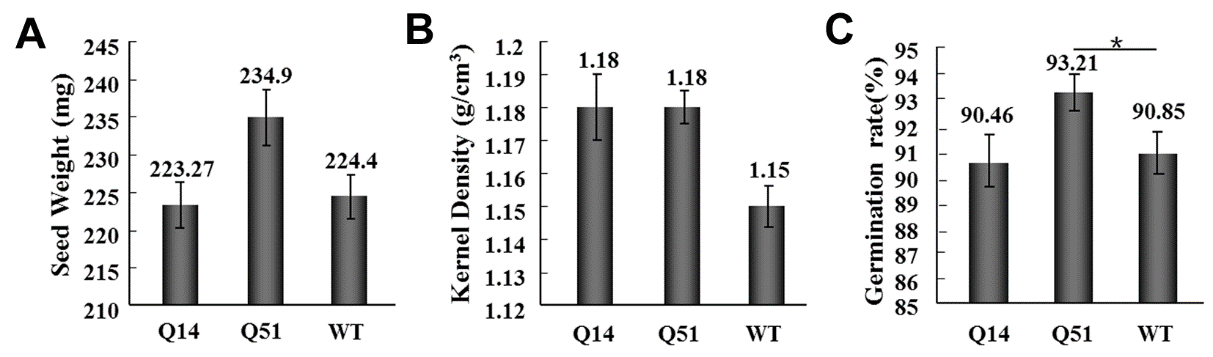


**Figure S2.** Agronomic quality analysis of WT, Q14 and Q51 kernels. (**A**), Seed weight; (**B**), Kernel density; (**C**), Seed germination rate. Student’s *t*-test was performed to evaluate the significance of difference between Q14, Q51 and WT control respectively (***** *p* < 0.05).

**Table S2.** Agronomic characters statistics of T_2_ generation.

| **Line** | **PH (cm)** | **EH (cm)** | **EL (cm)** | **KWPE (g)** | **ER** |
| --- | --- | --- | --- | --- | --- |
| 14 | 198.3 ± 18.8 | 78.4 ± 9.9 | 14.5 ± 3.1 | 117.6 ± 21.1 | 14–16 |
| 25 | 220.1 ± 23.4 | 104.4 ± 10.8 * | 9.5 ± 2.1 | 99.5 ± 17.9 | 12–14 |
| 28 | 217.2 ± 21.7 | 89.3 ± 7.8 | 10.7 ± 1.8 | 121.3 ± 18.4 | 12–14 |
| 47 | 195.5 ± 14.9 | 79.1 ± 11.0 | 11.7 ± 2.2 | 104.2 ± 11.6 | 14–16 |
| 49 | 231.9 ± 22.8 | 110.7 ± 13.7 ** | 12.9 ± 3.0 | 98.5 ± 13.9 | 12–14 |
| 51 | 189.4 ± 17.2 | 81.2 ± 11.3 | 12.5 ± 1.1 | 100.4 ± 14.8 | 12–14 |
| 55 | 201.5 ± 23.9 | 86.4 ± 10.2 | 13.4 ± 3.6 | 111.2 ± 21.8 | 14–16 |
| 60 | 224.1 ± 22.1 | 90.8 ± 9.0 | 14.6 ± 3.6 | 97.2 ± 9.9 | 14–16 |
| 62 | 183.3 ± 15.0 | 80.2 ± 7.9 | 14.3 ± 2.4 | 89.4 ± 7.1 | 12–14 |
| CT | 205.2 ± 23.1 | 85.7 ± 10.1 | 13.6 ± 2.4 | 103.2 ± 11.2 | 14–16 |

CT, Hi3027 segregation progenies with the same generation as transgenic lines. Plant Height, PH; Ear Height, EH; Ear Length, EL; Kernel Weight Per Ear, KWPE; Ear Rows, ER. Data were mean ± SD. Data were analyzed by One-way ANOVA (*p* < 0.05) and followed by *LSD* post test (* *p* < 0.05; ** *p* < 0.01).

**Table S3.** Agronomic characters statistics of T_3_ generation.

| **Line** | **PH (cm)** | **EH (cm)** | **EL (cm)** | **KWPE (g)** | **ER** |
| --- | --- | --- | --- | --- | --- |
| 14 | 173.2 ± 7.4 ** | 77.0 ± 11.4 | 11.6 ± 1.5 | 100.2 ± 12.3 | 14–16 |
| 25 | 187.3 ± 6.8 * | 82.3 ± 13.5 | 10.9 ± 2.4 | 98.4 ± 11.7 | 12–14 |
| 28 | 210.1 ± 18.2 | 88.2 ± 13.4 | 13.4 ± 2.0 | 92.0 ± 10.4 | 12–14 |
| 47 | 216.1 ± 17.7 | 85.5 ± 9.9 | 11.7 ± 0.9 | 87.9 ± 6.7 | 14–16 |
| 49 | 176.4 ± 13.2 ** | 75.6 ± 10.3 | 12.4 ± 3.1 | 81.1 ± 8.1 | 12–14 |
| 51 | 174.6 ± 8.9 ** | 76.9 ± 8.7 | 11.1 ± 2.3 | 90.8 ± 6.8 | 12–14 |
| 55 | 215.2 ± 20.6 | 88.7 ± 9.8 | 12.6 ± 4.1 | 93.2 ± 11.2 | 14–16 |
| 60 | 205.5 ± 16.1 | 79.1 ± 11.7 | 12.1 ± 1.4 | 88.3 ± 7.8 | 14–16 |
| 62 | 216.5 ± 17.9 | 85.1 ± 10.1 | 11.2 ± 2.5 | 74.2 ± 5.4 | 12–14 |
| CT | 221.7 ± 19.8 | 97.0 ± 12.4 | 12.4 ± 2.4 | 86.5 ± 3.6 | 14–16 |

CT, Hi3027 segregation progenies with the same generation as transgenic lines. Plant Height, PH; Ear Height, EH; Ear Length, EL; Kernel Weight Per Ear, KWPE; Ear Rows, ER. Data are mean ± SD. Data were analyzed by One-way ANOVA (*p* < 0.05) and followed by *LSD* post test (* *p* < 0.05; ** *p* < 0.01).

**Table S4.** Agronomic characters statistics of T_4_ generation.

| **Line** | **PH (cm)** | **EH (cm)** | **EL (cm)** | **KWPE (g)** | **ER** |
| --- | --- | --- | --- | --- | --- |
| 14 | 199.3 ± 16.5 | 76.3 ± 6.6 | 10.7 ± 1.5 | 90.4 ± 9.9 | 14–16 |
| 25 | 237.3 ± 14.8 ** | 108.5 ± 8.9 | 11.5 ± 0.8 | 94.3 ± 6.2 * | 12–14 |
| 28 | 180.8 ± 6.9 | 76.6 ± 8.3 | 15.2 ± 1.1 | 86.5 ± 2.4 | 12–14 |
| 47 | 195.6 ± 10.4 | 92.5 ± 10.1 | 10.5 ± 2.0 | 85.7 ± 1.3 | 14–16 |
| 49 | 200.1 ± 9.0 | 79.4 ± 7.3 | 10.9 ± 0.6 | 78.4 ± 4.1 | 12–14 |
| 51 | 182.6 ± 11.4 | 97.7 ± 6.7 | 12.7 ± 1.2 | 81.5 ± 5.0 | 12–14 |
| 55 | 227.4 ± 15.1 ** | 96.6 ± 11.2 | 12.9 ± 2,2 | 89.8 ± 3.9 | 14–16 |
| 60 | 189.7 ± 9.7 | 86.8 ± 8.6 | 10.4 ± 0.9 | 81.9 ± 5.2 | 14–16 |
| 62 | 208.5 ± 11.9 | 95.1 ± 5.2 | 11.9 ± 3.3 | 76.2 ± 3.8 | 12–14 |
| CT | 198.6 ± 8.9 | 68.3 ± 4.1 | 11.2 ± 1.4 | 82.8 ± 4.3 | 14–16 |

CT, Hi3027 segregation progenies with the same generation as transgenic lines. Plant Height, PH; Ear Height, EH; Ear Length, EL; Kernel Weight Per Ear, KWPE; Ear Rows, ER. Data are mean ± SD. Data were analyzed by One-way ANOVA (*p* < 0.05) and followed by *LSD* post test (* *p* < 0.05; ** *p* < 0.01).


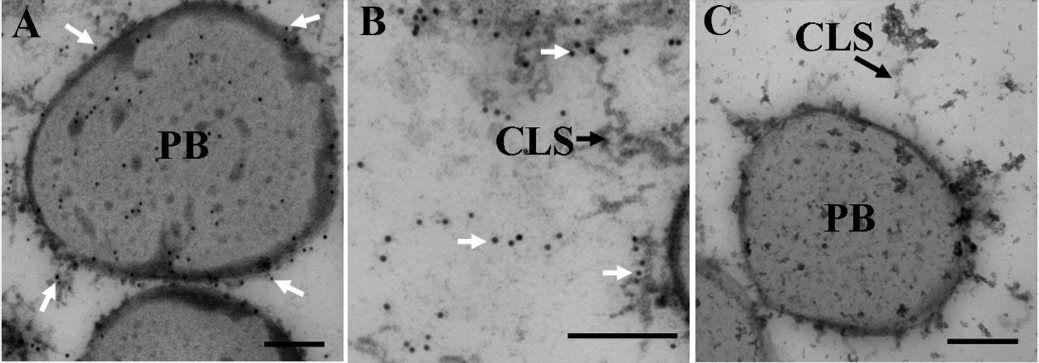


**Figure S3.** Immunogold labeling of SBgLR in Q14 endosperm. SBgLR mainly localized at the periphery of PB (white arrows in (**A**)) and the cytoskeleton-like structures (CLS) (white arrows in (**B**)); (**C**), No labeling was detected in Q14 endosperm when antibody was omitted in the experiment.
Bar = 200 nm in (**A**–**C**).
